# Beyond Corroboration: Strengthening Model Validation by Looking for Unexpected Patterns

**DOI:** 10.1371/journal.pone.0138212

**Published:** 2015-09-14

**Authors:** Guillaume Chérel, Clémentine Cottineau, Romain Reuillon

**Affiliations:** 1 Géographie-Cités, CNRS, Paris, France; 2 ISC-PIF, Paris, France; 3 Centre for Advanced Spatial Analysis, UCL, London, United Kingdom; Université Toulouse 1 Capitole, FRANCE

## Abstract

Models of emergent phenomena are designed to provide an explanation to global-scale phenomena from local-scale processes. Model validation is commonly done by verifying that the model is able to reproduce the patterns to be explained. We argue that robust validation must not only be based on corroboration, but also on attempting to falsify the model, i.e. making sure that the model behaves soundly for any reasonable input and parameter values. We propose an open-ended evolutionary method based on Novelty Search to look for the diverse patterns a model can produce. The Pattern Space Exploration method was tested on a model of collective motion and compared to three common a priori sampling experiment designs. The method successfully discovered all known qualitatively different kinds of collective motion, and performed much better than the a priori sampling methods. The method was then applied to a case study of city system dynamics to explore the model’s predicted values of city hierarchisation and population growth. This case study showed that the method can provide insights on potential predictive scenarios as well as falsifiers of the model when the simulated dynamics are highly unrealistic.

## Introduction

Writing models to explain emergent phenomena is trying to figure out what mechanisms at the local scale create the patterns observed at the global scale. Such models, often computer-based and requiring simulation, are keys to the scientific inquiry as soon as the system’s description involves a local and a global scale and it is not trivial to explain the global patterns from the local dynamics. They are increasingly developed in a wide range of disciplines from physics to biology, ecology, and social sciences. A long list of examples includes species evolution [1], problem solving in social insects [2], the dynamics of neural networks [3], social segregation [4], population dynamics [5], collective motion [6], and many more.

A classical model validation approach is to write a model of the local scale dynamics and interactions, calibrate it, run simulations, and compare the outputs to the data. These steps lead us to conclude whether the proposed model can explain the patterns observed in nature or not, and revise it as necessary [7–10]. But to say that the model can reproduce some patterns does not entail that it accurately reflects how they are produced in nature or society [11, 12]. A given global pattern can sometimes be explained by different models involving different local dynamics. An approach to validation based on mere corroboration is insufficient to build robust explanatory and predictive models. We need more rigorous practices. In this paper, we propose a method contributing to this need for simulated models by searching for unexpected model behaviours.

How can we test the validity of a model? We can make a useful detour by Karl Popper’s falsifiability principle. A theory yields specific statements which it allows or forbids. Allowed specific statements, when confirmed by empirical validation, corroborate the theory. Forbidden statements falsify it. Popper says that accumulating observations which corroborate the theory does not help in testing its validity. Consider the general statement “All swans are white”. Observing any number of white swans never discards the possibility of observing a black swan in the future. A single observation of a forbidden statement, on the contrary, immediately disproves it. It follows that one can only decide between competing theories by looking for their potential falsifiers.

**Fig 1:**
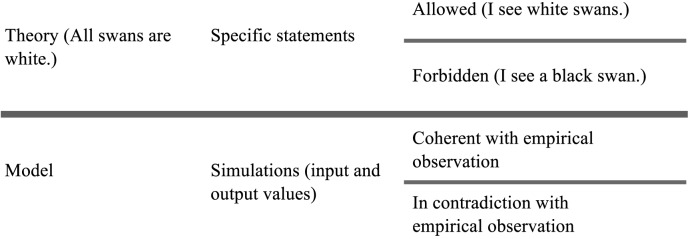
Modelling and simulation share a conceptual structure with Popper’s falsifiability argument.

Modelling emergent phenomena can be viewed as a specific case of the conceptual framework which Popper uses for his argument (Fig 1). A model is a general statement about how the world works. The model yields specific statements as individual simulations, i.e. associations of input and output values. The individual simulations can be partitioned into two sets: those which correspond to empirical observation, and those which contradict it. Members of the former corroborate the model, while members of the latter falsify it. It is of course important to ensure that a model is able to reproduce the patterns it is designed to explain. And it is equally important, following Popper’s argument, to look for simulations that may have the power to falsify the model, those which display an association of input values and output patterns which contradict data. We use the word *pattern* to denote a description of a simulation run. For example a simulation of a spatially explicit population dynamics model can be described in terms of population growth and spatial aggregation. All distinct combinations of values for these two variables are as many distinct patterns.

Models with high complexity can produce patterns that are unexpected even to the eye of the modeller. An unexpected pattern can either be a falsifier of the model if it is contradicted by subsequent empirical observation, or a prediction of the model if it is validated by it. To search for the unexpected patterns a model can produce in simulation is to search for both at the same time.

In the context of model building, searching for unexpected patterns should be thought of as one step in a broader iterative process. In this process, a model is designed, the pattern space explored, unexpected patterns (in particular falsifiers) used to revise the model assumptions, and we start over with exploring the pattern space with the revised model. The loop goes on until we get to a satisfying model.

The problem of searching for unexpected patterns is apart from the problem of calibration where some particular output is expected, since we do not know explicitly what to look for. We propose to approach it by looking more generally for the diverse patterns that our models can produce. The pattern obtained in a simulation depends on the model inputs (e.g. initial state) and the parameter values (controlling the model dynamics). Exploring the inputs can be interesting when studying what patterns can be produced from different initial conditions. Exploring the parameters is needed when there is uncertainty about their calibration (i.e. insufficient data). Input and parameter variables form together what we call here for simplicity the parameter space of the model (without loss of generality, inputs can be seen as model parameters, or equivalently variable parameters can be seen as model inputs). Thus while the exploration is driven within the pattern space in search for diversity, it is fundamentally a problem of parameter space exploration: how to explore the parameter space to find the various patterns, including those that are unexpected?

Such an exploration is no trivial task due to the common difficulties with computer-based models of complex systems: the high dimensionality of the parameter space, non-linearity between inputs and outputs, and stochasticity. High dimensionality (tens or hundreds of parameters) makes the parameter space prohibitively big to try out all possible parameter values (or a regular sampling in the case of real valued parameters). Non-linearity entails that interesting output values may correspond to very small regions of the parameter space, making them difficult to look for. Stochasticity makes it necessary to replicate the simulations. For models which take several seconds to run in simulation, a clever exploration method to reduce the number of simulations is necessary.

The active research field of experiment design aims at solving the problem of sampling vast parameter spaces. Some methods try to tackle the problem of dimensionality of the parameter space with a priori samplings, such as the well known Latin hypercube sampling or LHS (see examples of sampling methods and comparisons in [13–15]). Others try to solve it in an adaptive way, by choosing iteratively where to sample next based on information collected previously [16–18]. All start with the base problem of sampling efficiently (with a number of points that is tractable in simulation) the parameter space in order to get information about the relationships between the control parameters and the observables. They solve the problem by not exploring the parameter space exhaustively, depending on the information they do not need, given their particular objectives.

Our problem focuses on exploring the pattern space in search for unexpected patterns. We do not have any constraint about the parameter space. This transfers the problem of dimensionality from the parameter space to the pattern space. While it may be hard to reduce the number of free parameters of a model of complex system, modellers are free to choose what variables to measure on a simulation, i.e. how many dimensions the pattern space has. Typically, a 2 or 3 dimensional space has both the advantage of being tractable and easily visualised.

Evolutionary methods are well suited for this setting because they can perform a search through the parameter space based on information gathered in the pattern space by simulation, and do so without any a priori knowledge of the relationship between the parameter space and the pattern space. They were successfully used for model calibration when data about some parameters is lacking but there is an idea of global patterns to be expected [19–22]. Evolutionary calibration methods are all based on a priori ideas of the patterns one would like to obtain. The a priori target pattern can be based on data to reproduce an observed scenario. It can also be designed to represent an expected behaviour and used to assess the model’s ability to reproduce it (such as the V formation of flocks in [22]). An objective function is designed in terms of a distance between a model run and the target pattern, and evolutionary methods are used to tune the parameters to minimise it. Our problem is different in that, rather than targeting a particular pattern, we want to find the various ones a model can produce.

Miller [23] proposed a model breaking method using an evolutionary algorithms which looks for how far a model behaviour can depart from a calibrated behaviour when the parameters are kept within the vicinity of their calibrated values. Stonedahl and Wilensky used a similar approach for the purpose of sensitivity analysis [24]. This idea is getting closer to our objective of finding unexpected patterns, but has three limitations regarding this goal. First, it restricts the search to a small region of the search space. Second, it relies on a target pattern to which other produced patterns are compared. Third, like the calibration methods, it uses an objective-based search to maximise the distance from the target pattern. The objective-based search by nature progressively narrows the exploration towards a smaller region of the search space. While looking for unexpected patterns, we would rather not limit ourselves to just finding one, but instead look for all the different unexpected patterns we can find in the course of a single exploration. Rather than narrowing the search as objective-based methods do, we propose to continually widen the exploration in search for the diverse patterns a model can produce.

We propose to solve this problem with a new open-ended evolutionary algorithm based on Novelty Search [25, 26]. Novelty Search does not rely on an objective function. It generates successive populations of solutions to a problem by breeding the most novel individuals of the current population. The novelty of current individuals is measured with respect to the current population and an archive of past individuals, and reflects the novelty of patterns (not parameter values). Novelty Search continually drives the exploration of the parameter space towards areas which produce novel patterns. The method we propose here, called Pattern Space Exploration (PSE), is novelty search adapted to the exploration of agent-based and more generally computer-based models of complex systems. In standard Novelty Search [26], the novelty measure is based on the average distance of an individual to its k nearest neighbours in the pattern space. In PSE, novelty relies on a hit-map counter. The hit-map is a discretisation of the pattern space into regular cells, and we count the number of individuals which belong to each cell throughout the exploration. The novelty measure of an individual is based on the inverse value of its corresponding cell count. The less a cell has received individuals before, the more novel is an individual standing on it. This variation offers several advantages for our purpose. First, defining a distance measure in a multi-dimensional space is not a trivial task, in particular when the different dimensions represent unrelated variables. The hit-map approach alleviates this problem by suppressing the distance measure, and relying instead on the discretisation of the pattern space which is done independently for each dimension. Second, computing the k nearest neighbours is computationally costly (for example, finding the nearest neighbour in a KD-tree has complexity *O*(*log*(*N*)), where N is the number of individuals), and constitute a serious bottleneck in an evolutionary algorithm because it cannot be parallelised (while the individuals evaluations are independent and can be). The only operations needed with the hit-map are accessing or incrementing the counter at a given cell, and are done in constant time. Third, the hit-map naturally offers a measure of the exploration progress, the *volume discovered* in the pattern space, approximated by counting the number of cells which counters are positive.

In this paper we present PSE and two sets of experiments. The first one was designed to test the method’s efficiency in exploring the pattern space of a model, i.e. in finding a wide range of different patterns. We used a model of collective motion and compared PSE to common a priori sampling methods (LHS, regular grid, Sobol). Collective motion is a well studied phenomenon in physics and the different patterns that can be exhibited is well described. The second experiment was a real modelling scenario with a recently developed model of system of cities—MARIUS (Model of Agglomerations within Imperial Russia and the (former) Soviet Union) [27]. We used PSE to explore thematic questions regarding the possible outcomes that the model is able to simulate. This experiment illustrates one iteration of the iterative modelling process proposed above (the pattern space exploration and the analysis of unexpected patterns to revise the model’s assumptions). The following sections present the algorithm and the two experiments along with their results.

## Algorithm description

The Pattern Space Exploration method (PSE) is an open-ended evolutionary algorithm. It is composed of the common building blocks of evolutionary algorithms (see algorithm 1): a population of individuals whose genomes each encode a particular element of the search space, a selection method to select individuals from which a new population will be created, a crossover and mutation mechanisms to create a new individual from previously selected ones, and an elitism mechanism to filter all the individuals (newly created and the old population) to be kept and form the new population. As the algorithm progresses, unwanted individuals are discarded and forgotten as new ones are added to the population. An archive is used to store information about past populations. Each of these building blocks can be specified in different manners in order to build different evolutionary algorithms. Below, we will describe how we designed each building block to implement the PSE method.

      1 Generate *μ* genotypes by sampling the genotypic space randomly

      2 Evaluate them

      3 **while** *stop condition not reached* **do**

      4 **for** *i* ← 1 **to** *λ*,*s the number of new individuals to create* **do**

selection    5   Select 2 individuals from the population

crossover      6   Create a new genotype by crossover from the 2 selected individuals’ genotypes

mutation    7   Mutate the new genotype

      8  **end**

evaluation  9 Compute the offsprings’ phenotypes

elitism    10 Filter the individual of the whole population (parents and offsprings) based on their phenotype

      11 **end**

**Algorithm 1:** General scheme of an evolutionary algorithm

**Fig 2:**
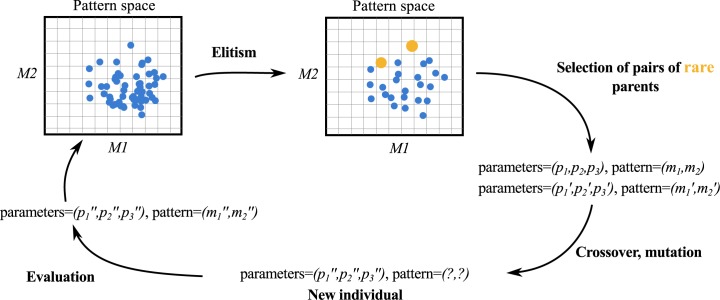
General scheme of the Pattern Space Exploration method. M1 and M2 represent the dimensions of the pattern space. The p_i_ represent individual parameter variables, and the m_i_ represent pattern variables. The pattern space is discretised into cells and rare (novel) parents are selected based on the hit count of their corresponding cell. New individuals are generated by crossover and mutation and evaluated to get their pattern. Newly evaluated individuals increment the hit count of their respective cell. All individuals (parents and offsprings) are filtered at the elitism stage so only one per cell remains, randomly picked for each cell.

PSE is illustrated in Fig 2. The algorithm starts by generating individuals with random genomes representing parameter values (the *new individual* stage in the figure). For each, a simulation is run and the pattern is measured (evaluation). This results in as many patterns as generated individuals. At the elitism stage, individuals are filtered to keep only those that are significantly different from one another with respect to their patterns. Pairs of individuals are selected based on how rare their patterns are relative to the current population and past ones (rarity is PSE’s equivalent of the novelty measure). Each element’s genome from each pair is recombined to form a new genome, which is then further mutated. A new generation of individuals is thus created, and the loop continues. The main idea is that by selecting parents whose patterns were rarely observed previously, we increase the chances to find patterns yet undiscovered.

### Individuals (genotype, evaluation, phenotype)

An individual is described by a genome and a phenotype. Its genome encodes an element of the search space. The phenotype of the individual is computed given its genotype through evaluation. The phenotype thus reflects the behaviour of the individual.

In PSE, a genotype encodes a value for each parameter. An evaluation corresponds to a simulation run with the parameter values taken from the genome. A phenotype encodes a pattern measured on the simulation run.

Here, both the genotypic and phenotypic spaces are multidimensional real spaces, respectively ℝ^*K*^ and ℝ^*M*^, where K is the number of parameters of a model, and M is the number of descriptive variables of a simulation. The restriction to real values is done for simplicity. The experiments below seamlessly use integers in some of the parameters dimensions by casting them to doubles to construct the genotypes, without any alteration of the algorithm. The use of categorical (non-ordinal) or more complex parameter types would require using other more appropriate random sampling methods (for population initialisation), as well as crossover and mutation operators than those used here. In the pattern space, the algorithm can also deal with integers and categorical values by simply mapping them to real values. More complex types might pose scalability issues if they implicitly add dimensions to the pattern space, and might be difficult to discretise (see archive below).

When the model dynamics or initialisation are stochastic, it is necessary to replicate each simulation for statistical robustness. In this case, the median over the replications is used for each of the M phenotypic variable.

### Initialisation

The first generation of *μ* individuals is created with random genomes, by drawing uniformly a value in each of the model parameter domain.

### Archive

PSE uses a hit map archive to keep a trace of how much the different regions of the phenotypic space have been explored. This information is used at the selection stage to select rare individuals, and at the elitism stage to avoid the accumulation of too many individuals in the same region of the space (see below). The archive consists in a user-specified discretisation of the phenotypic space along each dimension, thus forming a set of M-dimensional cells covering the phenotypic space. The discretisation is provided as a step size for each phenotypic dimension. The resulting discretisation in each domain is “anchored” on 0 (a step size of 1 places the discrete bounds at … -2, -1, 0, 1, 2 …). Each phenotype can be naturally located in its corresponding cell. Each cell has an associated hit count summing the number of individuals’ phenotypes which were located in the cell throughout the evolutionary process. The hit map is updated between the evaluation stage and the elitism stage. The hit map is encoded as a sparse dictionary where keys represent cell positions and the values represent the hit counts. Keys are added to the hit map when new phenotypes are found. Any key not present in the hit map implicitly has a value 0. This allows to deal with the discretisation of an infinite space without having to set min and max bounds. Bounds can be set artificially to focus the exploration on a certain region, as explained in the experiments below.

### Selection

Traditionally, classical objective-based evolutionary algorithms rely on a measure of fitness to select the individuals. The fitness reflects the quality of an individual, that is how well it performs regarding the objective. The selection stage then consists in choosing fit individuals in order to move the search towards good solutions. Here however, our objective is to find diverse phenotypes. There is no notion of fitness. In PSE, we select the individuals whose phenotypes are rare. By selecting these, the exploration will focus on the areas of the phenotypic space that were yet little explored. The rarity of a phenotype is given by the inverse of the hit count of the corresponding cell in the hit map archive.

Selection is performed as a binary tournament. Two individuals are picked at random in the whole population, then the individual with the rarest phenotype is kept. The tournament is performed every time there is an individual to select. Thus it is performed twice to select the 2 parents of a new individual to be created.

### Crossover and mutation

We used the adaptive Cauchy mutation [28, 29] and SBXCrossover [30].

### Elitism

The elitism stage limits the growth of the population size. After the evaluation of the new offsprings, each of them is attributed to an archive cell corresponding to its phenotype. In cells where there is more than 1 individual (considering new offsprings as well as individuals of the parent population), one is selected randomly and the others discarded. The individuals that remain form the new population. This is comparable to a *plus* selection scheme, denoted (*μ* + *λ*) where *μ* is the population size and *λ* the number of offsprings, with the particularity that the population size grows since a new individual located in a cell with a 0 hit count can be incorporated into the population without replacing any other.

### Stop condition

PSE runs continually. It is up to the user to decide when to stop the exploration. The decision can be based on the evolution of the number of distinct patterns (or volume discovered, i.e. the number of cells in the archive whose hit-count is greater than 0), for example, when the rate of pattern discoveries slows down. Caution must be taken about interpreting this as a sign that all or most patterns were discovered. There are two possible interpretations to the slowing down of the rate of discoveries: either the model cannot exhibit any other pattern (according to the discretisation scheme) than the ones already discovered, or the remaining patterns are located in a region of the search space that is very hard for the exploratory process to access. The latter possibility is the consequence of non-linearity between the parameter space and the pattern space. This uncertainty is inherent to evolutionary algorithms, but it is also what constitutes their strength: the ability to continually look for better solutions (in the case of objective-based evolutionary algorithms) or to search for diversity (in the open-ended objective-less case) in absence of any a priori information about the relationship between the search space and the output or pattern space. They are best suited for the cases where little such information is available to the modeller, which is common in computer-based models of complex systems.

### Parallelism

Evolutionary algorithms are naturally adapted to parallel computing since individuals evaluations are independent from one another. In the experiments below, we used two different parallelisation strategies: a parallelised steady state model and an island steady state model. (The first strategy made easier to control and record the number of model evaluations performed in time, but was slower on the EGI grid (see section Software and hardware infrastructure). The second strategy was more efficient at exploiting the grid’s computing resources and was thus used for the most computationally intensive experiment.)

With the standard steady state model, only one individual is created at each generation. For parallelisation, we had a pool of *λ* individuals continually evaluated in parallel. Every time an evaluation is done, the corresponding individual and the current population go through the stages of elitism, selection, crossover and mutation to generate a new individual fed back to the pool for evaluation. This is close to the standard steady state model, (*μ* + 1), with the difference that when a new individual is created, some of the individuals that were created before it are still in the evaluation pool and weren’t yet sent back to the population.

The island steady state model works by splitting the global evolution into several smaller evolutions (islands) which can be run in parallel. A global population and global hit-map are created on the main computing node. When an island is started on a distant node, it is initialised with a random subset of the global population. It then runs the complete PSE algorithm with the standard steady state model (*μ* + 1) for a fixed amount of time. When it completes, its final population is used to update the global hit-map and is merged back into the global population with the same elitism operator as described above. Subsequent islands are then initialised with populations sampled from the updated global population. A fixed number of islands are run in parallel. When one finishes, a new one is started.

### Usage

To use PSE on a given model, the following information must be specified:

- which model parameters constitute the genotypic space (some model parameters may be excluded from the search space by fixing their values or initialising them randomly),
- a set of measures describing the simulation output, constituting the phenotypic space
- bounds on the parameters,
- discretisation step size on the phenotypic dimensions,
- *μ*, the size of the initial population,
- the probability of reevaluating a selected individual without performing any crossover and mutation,
- if the parallelised steady state model is used, *λ*, the size of the evaluation pool,
- if the island steady state model is used, the number of islands run in parallel and the population size for each island.

## Software and hardware infrastructure

PSE was developed in Scala within the MGO library, an open source library for evolutionary algorithms. The source code is available as a git repository (https://github.com/openmole/mgo).

The models presented below were also developed in Scala within the Simpuzzle project, and also available as a git repository (https://github.com/ISCPIF/simpuzzle).

The simulation experiments described below were deployed on the European Grid Infrastructure through the *biomed* virtual organisation.

Parallel deployment of the simulations on the grid was performed with OpenMOLE (http://www.openmole.org/) [31], an open source software developed at ISC-PIF dedicated to the execution of simulation experiments on distributed computing environment (multicore machines, clusters, grids). MGO is tightly integrated to OpenMOLE, which allowed to easily use PSE within OpenMOLE to drive the experiments.

The script files, models and instructions necessary to reproduce the experiments presented below are available in the github repository https://github.com/ISCPIF/PSEExperiments.

Developers interested in using PSE without OpenMOLE should refer to the MGO library mentioned above. Refer to the last section of the README file in the PSEExperiments github repository for instructions to get started.

## Test case: collective motion

The study of collective motion, or flocking, is the study of how self-propelled and locally interacting particles can organise to move together as a whole. What is particularly intriguing about these systems is their capacity to produce collective motion and complex collective behaviours like avoiding obstacles without any leader. These phenomena have been extensively studied by physicists starting with Vicsek [32, 33], and others since then, both analytically and in simulation [34].

The literature contains precise descriptions of the collective behaviour of flocking models [6]. Knowing what behaviours were to be expected made it a good test case for our method.

We tested our Pattern Search Exploration method on the model described below. In order to compare the method to some famous a priori experimental designs, we also used a LHS, a regular grid sampling, and the Sobol sampling method.

### The flocking model and simulation

We implemented a version of Reynolds’ famous flocking model [35] inspired by the flocking model available in Netlogo’s model library [36, 37].

The entities called boids are located in a 2D toroidal environment. Each is described by a position and a heading. For an environment of width E_W_ and height E_H_, the boid i has a position (*x*_*i*_, *y*_*i*_) ∈ [0, *E*_*w*_) × [0, *E*_*h*_) and a heading *h*_*i*_ ∈ [−0, 2*π*).

Each boid tries to avoid collision with nearby flockmates while matching their heading and staying close to them. The collision avoidance mechanism has precedence over the other two.

All boids positions and headings are updated simultaneously from one time step to another, avoiding any effect of the order in which new states are computed. At each time step, a new heading is computed for each boid based on its current heading and the state of its neighbours, i.e. other boids within radius *rVis*. Boids then step forward by a fixed distance *stepSize*.

If the distance of boid i to its nearest neighbour j is less than *minSep*, then i only tries to avoid collision. It turns away from j’s heading, with a maximum angle turn of *maxTurnS*. Its new heading is given by:

$$\begin{array}{c} h_{i}\left(t+1\right)=h_{i}\left(t\right)+T\left(A\left(h_{i}\left(t\right),h_{j}\left(t\right)\right),maxTurnS\right). \end{array}$$

Where A(h_i_, h_j_) gives the angle from h_i_ to h_j_ such that (*h*_*i*_ + *A*(*h*_*i*_, *h*_*j*_)) *mod* 2*π* = *h*_*j*_. The function T keeps the turn angle absolute value less than or equal to *maxTurnS*:

$$\begin{array}{c} T(a,m)=sign(a)\times min(abs(a),m),\;m\geq 0. \end{array}$$

If boid i has no neighbour that is too close, it will simultaneously try to match its flockmates average heading and turn towards their average position. In each component (match flockmates headings and turn towards flockmates) the turn is limited by a maximum angle respectively of *maxTurnA* and *maxTurnC*:

$$\begin{array}{ccc} h_{i}\left(t+1\right)=h_{i}\left(t\right) & + & T({\left\langle h_{j}\left(t\right)\right\rangle }_{j∼i},maxTurnA)\\ & + & T(\theta \left({\left\langle x_{i}-x_{j}\right\rangle }_{j∼i},{\left\langle y_{i}-y_{j}\right\rangle }_{j∼i}\right),maxTurnC). \end{array}$$

*j* ∼ *i* is the set of particles j within distance *rVis* of i. *θ*(*x*, *y*) gives the *θ* component of the point (*r*, *θ*) in polar coordinates that corresponds to the point (x,y) in Cartesian coordinates.

The population is constituted of N_b_ boids which are initialised randomly at the beginning of a simulation.

The model has 9 parameters (see Table 1). In the following simulations, the four first are fixed to the values displayed in the table. The model has thus five free parameters which will constitute the search space.

**Table 1:** Parameters for the flocking model.

Parameter	Value	Description
E_W_	32	Environment’s width
E_H_	32	Environment’s height
N_b_	128	Number of boids
*stepSize*	0.05	Boids speed
*rVis*		Neighbourhood radius (vision)
*minSep*		Minimum separation distance allowed to avoid collision
*maxTurnS*		Maximum turn allowed to avoid collision
*maxTurnA*		Maximum turn allowed to align with flockmates
*maxTurnC*		Maximum turn allowed to get closer to flockmates

Simulations are run for 1000 time steps, both to give reasonable time for flocks to form and avoid too long simulation time.

### The pattern space

Grégoire, Chaté and Tu [6] proposed five qualitatively different behaviours a flock can exhibit: gas, moving solid, stationary solid, moving liquid and stationary liquid. In the gas phase the particles do not coalesce into flocks but move independently. In the solid phase, particles keep a fixed position relative to one another in time. In the liquid phase, particles move relative to one another within the flock, as seen in schools of fishes and flocks of birds. We used these five kinds of behaviours as landmarks to test our method.

They use three order parameters to describe a flock’s behaviour, described below, which we use to constitute the pattern space. They describe the formation of flocks, liquid/solid phases, and the flocks’ velocity.

The gas phase corresponds to particles not forming a cohesive flock. “A cohesive flock is one for which n, the size of the largest cluster, is of order N, the total number of particles” [6]. As the first dimension of the observable space we can take the size of the biggest cluster. The clusters were computed after 1000 iterations of the model. They are formed by connecting together the particles which are within a distance *rVis* of one another. The clusters are the connected components of the resulting graph.

The solid and liquid phases differ by how much the particles move relatively to one another during the simulation, that is how the distance between initially neighbouring entities at time t varies at t + T_d_ for large T_d_. To capture this phenomena, Grégoire, Chaté and Tu [6] use the following relative diffusion measure:

$$\begin{array}{c} \Delta \equiv {\left\langle \frac{1}{n_{i}}\sum_{j∼i}(1-\frac{r_{ij}^{2}\left(t\right)}{r_{ij}^{2}\left(t+T_{d}\right)})\right\rangle }_{i,t}, \end{array}$$

where n_i_ is the number of neighbours of particle i at time t, and r_ij_ the distance between particles i and j. In *j* ∼ *i*, the js are all the neighbours of i in the Voronoi sense. Δ ∼ 1 in the liquid phase and Δ ∼ 0 in the solid phase.

Rather than averaging over all t, which is computationally costly, we limited t to 5 different values towards the end of the simulation, i.e. *t* ∈ {1000 − *T*_*d*_,1000 − 2*T*_*d*_,1000 − 3*T*_*d*_,1000 − 4*T*_*d*_,1000 − 5*T*_*d*_}. The environment being toroidal, too large values of T_d_ would have compromised the distance measures. When two initially neighbouring particles move in different directions, their distance starts shrinking after growing beyond half the environment’s linear size. We set T_d_ so that an individual could not travel more than a fourth of the environment linear size, *T*_*d*_ = *min*(*E*_*W*_, *E*_*H*_)/(4 × *stepSize*). It remains possible, due to the fact that neighbours are usually separated by a non null distance, that initially neighbouring individuals moving away from each other will cross that distance, leading to smaller Δ than should be observed. This pattern however should only concern cases where boids do not remain in cohesive flocks, i.e. the gas phase. The problem should thus not interfere when cohesive flocks do form, which is when Δ will be of interest to distinguish the liquid phase from the solid phase.

Finally, in order to distinguish between the stationary and moving phases, we measured the average velocity V of the particles towards the end of the simulation. The velocity at t was measured by the average distance between each individual’s position at t and t + T_v_, where *T*_*v*_ = *min*(*E*_*W*_, *E*_*H*_)/(2 × *stepSize*).

$$\begin{array}{c} V={\left\langle \frac{1}{N}\sum_{i}d_{i}\left(t,t+T_{v}\right)\right\rangle }_{t}, \end{array}$$

where d_i_(t, t + T_v_) is the euclidean distance travelled by particle i between t and t + T_v_. Here again, *t* ∈ {1000 − *T*_*v*_,1000 − 2*T*_*v*_, 1000 − 3*T*_*v*_,1000 − 4*T*_*v*_, 1000 − 5*T*_*v*_}.

### Pattern Space Exploration configuration

The variables constituting the genotypic space are the free parameters of the model, and their domain bounds are given in Table 2. The boids positions and headings are initialised randomly. Individual evaluation was done with 100 replications. The phenotype is the median of each 3 variables of the pattern space over the replications. Table 3 gives the discretisation step size of their respective domains. Additionally, the domain of exploration for Δ was bounded with a minimum of -1. Simulations whose Δ value were beyond it were assumed to have Δ = −1. Since Δ itself has no lower bound, this artificial bound was set to avoid pushing the exploration below -1.

**Table 2:** Bounds on the flocking free parameters domains.

	*rVis*	*minSep*	*maxTurnS*	*maxTurnA*	*maxTurnC*
min	0	0	0	0	0
max	32	32	*π*	*π*	*π*

**Table 3:** Discretisation step size of the observables for the flocking model. Min and max values give the theoretical bounds for each observable. Between parentheses is given an artificial bound that was set for Δ in the experiments. Any lower value encountered in a pattern were set back to -1 in the phenotype. This trick allowed us to focus the exploration on regions of interest.

	Biggest cluster size	Relative diffusion (Δ)	Average velocity (V)
step	1	0.2	0.1
min	1	−∞ (-1)	0
max	128	1	0.5

The initial population size parameter *μ* was set to 5000, and the probability of reevaluating an individual (rerun all replications on a selected point without crossover or mutation) to 0.01. We let the exploration run until the total number of evaluations reached 100000 (i.e. 10 million simulations, since there are 100 replications per evaluation). The parallelised steady state model was used. The size of the evaluation pool *λ* was set to 5000.

In order to test the robustness of the method regarding stochasticity, the exploration was replicated 10 times.

### Benchmark of PSE against a priori samplings

We compared PSE’s ability to reveal the diverse patterns a model can produce, using the flocking model, to common a priori sampling methods: a regular grid, LHS and Sobol’s sampling. Those produce points in the parameter space. As with PSE, for each point, 100 replicated simulations were run, and the median for each pattern variable was computed.

#### Regular grid

In the regular grid sampling, each parameter domain was sampled at regular intervals. A sampling was then produced by all the combinations of values for each parameter. We produced 6 samplings respectively containing i^5^ points, i varying from 4 to 10 being the number of samples per parameter domain.

#### LHS and Sobol

These methods are multidimensional low-discrepancy sampling methods. LHS is stochastic and Sobol is deterministic. For each, we generated 10 samplings respectively containing i × 10^4^ points, with i varying from 1 to 10. The LHS sampling was replicated 10 times.

### Results

#### Patterns discovered with PSE

**Fig 3:**
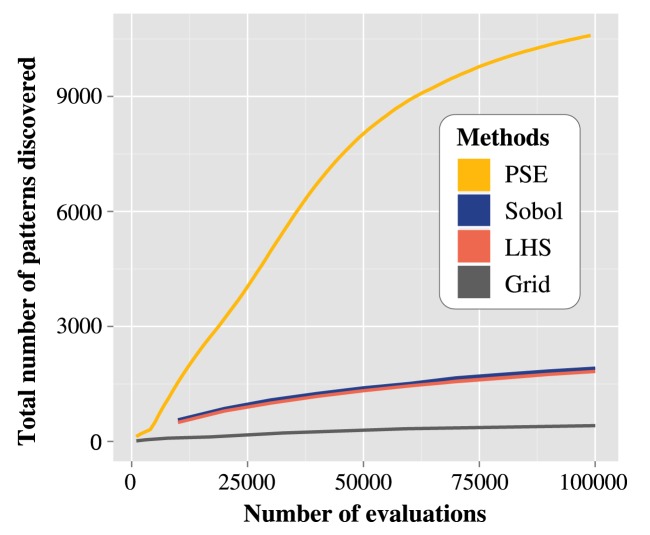
Comparison of the rate of pattern discovery in time for the flocking model. Number of patterns discovered as a function of the number of evaluations with the different methods tested (the model initialisation is stochastic, therefore 1 evaluation = 100 simulations). For PSE and LHS, averages over 10 replications are represented. Standard deviations were too small to be represented.

The progression of the number of patterns discovered slows down after 60000 evaluations (Fig 3). Out of the 100000 evaluations, 95% of the patterns were discovered within the 81400 first evaluations on average (sd. 663).

**Fig 4:**
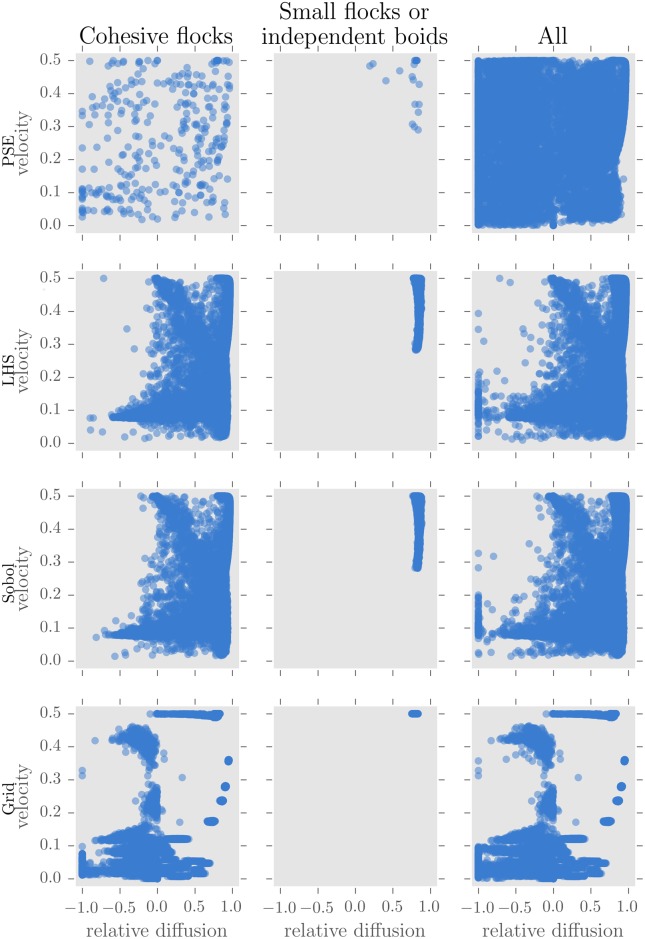
Patterns discovered by PSE and 3 a priori samplings using the flocking model. Plots on the left show the results of simulations where big clusters of at least 125 boids formed, plots in the middle those where the clusters were constituted by 2 individuals at most, and plots on the right show the results of all simulations.

The five different kinds of patterns enumerated previously were found. In Fig 4, the top row shows the patterns found by PSE. The left column shows patterns where large cohesive flocks form, and the middle column those where particles move independently or in small groups. The latter correspond to the gas phase. In the former, we can distinguish the 4 other kinds of patterns.

In the top left hand plot, patterns with a relative diffusion (Δ) close to 0 corresponds to the solid state, and those close to 1 correspond to the liquid state. The exploration also found negative values which denote flocks contraction. On the velocity axis, we can distinguish between moving (0.5 is the velocity of a particle moving straight) and stationary states. We can see that the exploration found a range of patterns from stationary to moving and from liquid to solid to contracting motion.

These results where similar across the 10 replications of PSE (see S1 Fig).

#### Comparison of PSE to other a priori samplings

**Fig 5:**
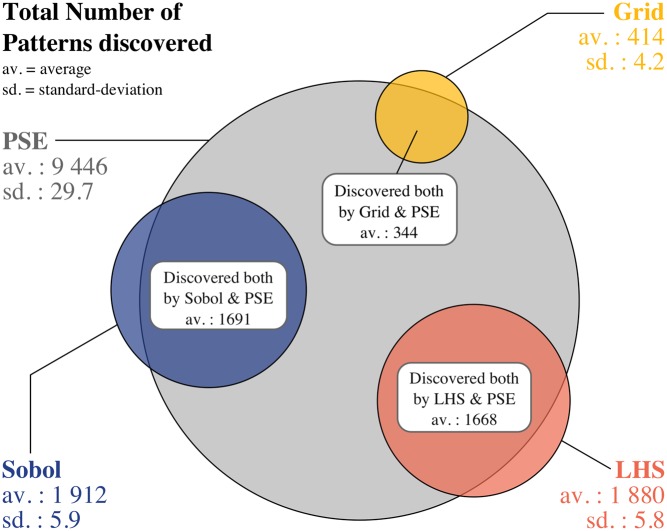
Number of patterns discovered by PSE and each compared method using the flocking model. Quantities are averaged over the experiments replications for stochastic methods. Intersections between the a priori samplings are not shown.

As seen in Fig 3, PSE was the fastest and most efficient method in terms of number of distinct patterns discovered. PSE found many patterns which were not discovered by the other methods, and only few were discovered by the other methods that were not discovered by PSE, as seen in Fig 5.

Fig 4 offers a visual comparison of the patterns discovered by the different methods. PSE found a bigger variety of behaviours for cohesive flocks as well as small flocks. The LHS and Sobol samplings had trouble finding cohesive flocks with negative relative diffusion and high velocity (see S2 Fig for the results of each LHS replication). The regular grid sampling also missed many patterns of positive relative diffusion which were largely discovered by all other methods.

## Use case: exploration of the patterns of hierarchisation and population growth of a system of cities with the model MARIUS

In an empirical case study about the evolution of the system of cities in the post-Soviet space, the method proved useful to explore the behaviour of a large agent-based model (in terms of the number of agents (1145) and running time), and to give an answer to some thematic and theoretical questions regarding the evolution of this system as modelled within the model MARIUS.

### Thematic incentives for Pattern Space Exploration

The objects of investigation of the social sciences in general, and the geographical study of systems of cities in particular, face the problem of the impossibility to run experiments in vivo and to reproduce an observed evolution under different conditions. However, thinking of societies as complex systems imply to consider parts of their trajectories and configurations as contingent, and to acknowledge that the observed outcome is just one out of many different possible scenarios [38, 39]. We could even say that “to know a society and a geography is to know how it could be different than it is.” [40]

This is why computer-based modelling and simulation in silico provide interesting tools to explore the possible outcomes of a given society and geography under changing sets of conditions [41], and in particular, this is why the PSE tool is a good way to explore the diversity of possible trajectories that the modelled systems can reach according to changes in the parameters.

In the case of MARIUS, which aims at simulating interactions and growth patterns of cities of the Former Soviet Union, we wanted to explore the possibility of unprecedented situations. Almost all the systems of cities that we are able to monitor via census data in the world since the invention of censuses are growing systems, and most of them have exhibited a tendency towards hierarchisation—the accentuation of the degree of inequality regarding cities populations (a city population and its rank in the system are related by a power low, meaning hierarchised systems have a few large cities and many small ones, as in the canonical Zipf’s Law) [42]. However, post-Soviet cities, and Russian cities even more, have been experiencing an unprecedented process of demographic shrinkage since the fall of the USSR, while persisting in the hierarchisation of the system [43, 44].

MARIUS was developed to reproduce the particular structure of the Soviet system of cities with a parsimonious set of generic rules. A study showed its ability to reproduce the cities historical hierarchical structure evolution from 1959 to 1989 [27], i.e. hierarchisation of the cities in a context of population growth. The corresponding model parameters calibrated values are shown in Table 4. In this paper, we use PSE to find out which possible degrees of hierarchisation and growth the model can produce. Four main types of outcomes can be expected, the first three of them were already confirmed by historical data as mentioned above:

- hierarchisation in a growth context (e.g. United-States in the XXth century, Russia between 1959 and 1989),
- hierarchisation in a shrinkage context (e.g. Russia in the 1990s and 2000s, Germany in the 2010s),
- equalisation in a growth context (e.g. China in the 1960–70s),
- equalisation in a shrinkage context.

**Table 4:** MARIUS parameters. Bounds for the experiments and calibrated values from [27] reproducing Russia’s cities hierarchisation and growth during the late XXth.

parameter	min	max	calibrated value
*economicMultiplier*	0	100	0.3438093442
*sizeEffectOnSupply*	1	2	1.0017563880
*sizeEffectOnDemand*	1	2	1.0792607803
*wealthToPopulationExponent*	0	2	0.3804356044
*distanceDecay*	0	10	0.6722631615
*populationToWealthExponent*	1	10	1.0866012754
*bonusMultiplier*	0	1000	197.9488907791
*fixedCost*	0	1000	0.2565248068

### MARIUS

The full ODD description of the model is available in the S1 Appendix and on github (https://github.com/ISCPIF/marius-method/blob/master/ODD-MARIUS.pdf). The following is a brief overview.

The MARIUS model includes basic economic rules of interurban interactions. The system is made of collective agents: the 1145 agglomerations of more than 10 000 inhabitants in 1959, localised via actual latitudes and longitudes in former Soviet Union. It is initialised with empirical populations and a fictive wealth quantity estimated as a superlinear function of population (i.e. with the parameter *populationToWealthExponent* being greater or equal to 1).

At each step:

1. Each city updates its economic variables according to its population: supply and demand, modelled as superlinear scaling quantities. Two parameters, *sizeEffectOnSupply* and *sizeEffectOnDemand* shape the non-linear advantages in productivity and consumption per inhabitant according to the city size. The higher they are, the more big cities are economically productive and consumptive per capita. The parameter *economicMultiplier* adjusts these quantities to a fictive unit of wealth.
2. Each city interacts with the others according to the intensity of their interaction potential (a function of the masses of the two cities and the distance between them, depending on a parameter *distanceDecay* representing the role of distance on interaction potential). For two distinct cities A and B, an interaction consists in confronting A’s supply to B’s demand, resulting in a potential transaction of goods sent from A to B.
3. Potential interactions are selected if the potential exchange exceeds a profitability threshold *fixedCost* representing the fixed costs of interurban exchanges non proportional to distance (the 4T costs of [45]: transaction, transportation, temporal, tariff and non-tariff costs). This mechanism reflects the fact that pairs of small and distant cities are not supposed to interact for very small amounts. That way, cities do not necessarily exchange with all other cities in the system.
4. Each city updates its wealth according to the results of the transactions in which it was committed and gets an economic impulse from the amount and diversity of exchanges. This reward of interactions relies on a parameter called *bonusMultiplier*, which represents the magnitude of exchanged externalities (information, knowledge, acquaintance, etc.).
5. Each city updates its population according to its new resulting wealth. The conversion is differentiated according to the size of the city’s population, with a scaling exponent *wealthToPopulationExponent*. The economic literature is not definitive about the range of values for this parameter [46, 47]. Below a value of 1, the same gain in wealth generates proportionally more population growth in smaller cities. Over 1, the same gain in wealth is demographically greater for larger cities.

The date used for initialisation is 1959, as it refers to a census date that opens a period of stability in the Soviet Union and a generic evolution of urbanisation. Each simulation runs for 30 time steps corresponding to one year each, therefore leading to a simulated 1989.

### The pattern space

The pattern space for MARIUS is 3-dimensional. It was designed to reflect first of all the hierarchisation and population growth of the system. Hierarchisation was measured as the slope of the size-rank distribution at the end of the simulation, i.e. the exponent of the power law relating a city size to its rank in the system. Population growth was measured as the difference between the final total population and the initial one. To distinguish between simulations where population growth is monotonic from those where it is more complex, we added as a third variable the number of times the sign of the population growth changed during the simulation.

### Pattern Space Exploration configuration

The model has eight free parameters. Their domain bounds are given in Table 4.

The model is deterministic. The phenotypic space corresponds to the pattern space described in the previous section. Each dimension is discretised according to Table 5. The phenotypic variables were bounded to focus the exploration on intervals of interest. The population growth was upper bounded to 500 million, the inversion count was upper bounded to 3, and the rank-size slope (hierarchisation) was lower bounded to -10. Simulations whose pattern values went beyond these were assumed to have a phenotypic value of the corresponding bound.

**Table 5:** Pattern variables for MARIUS. The step column gives the discretisation step size. Min and max values give the theoretical bounds for each observable. Artificial bounds were set on some parameters to focus the exploration on regions of interest. They are given inside parentheses. Calibrated values are from [27], reproducing Russia’s cities hierarchisation and growth during the late XXth.

	step	min	max	calibrated value
Rank-size slope	0.1	−∞ (-10)	0	-1.151
Population difference	5000	-0.83544 × 10^8^	+∞ (500 × 10^8^)	65554000
Inversion count	1	0	28 (3)	0

First, we ran a “long run” experiment where we let PSE run for a total of approximately 36 million model evaluations. We executed a total of 200000 islands for two hours each, distributed on 5000 computing units. One model evaluation takes about 40 seconds. The whole run took 400000h in cumulative computation time. The actual computation time was reduced to about a week thanks to the computational capabilities of the EGI grid. In this first experiment, the exploration was not replicated. To compare the rate of discovery between PSE and Sobol, we performed a second experiment involving shorter explorations using the parallel steady state method. PSE was replicated 10 times and each exploration (the 10 PSE replications and the Sobol sampling) was run for 3.5 million model evaluations.

### Results

**Fig 6:**
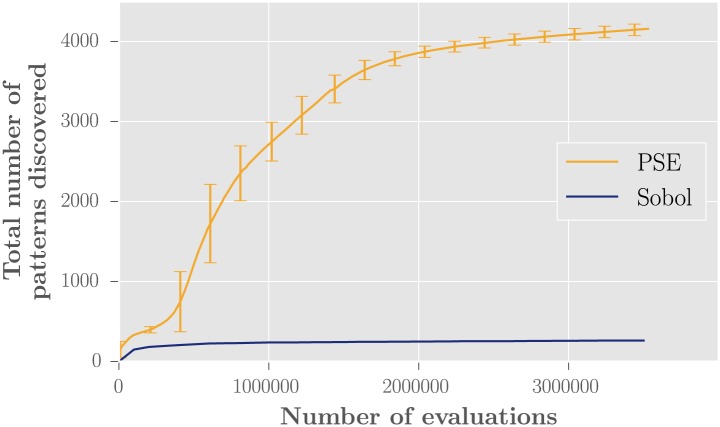
Number of patterns discovered by PSE and Sobol using the model MARIUS. Abscissa gives the number of model evaluations. The PSE curve displays the average and standard deviation over 10 replications. PSE was much more efficient than the Sobol sampling.

**Fig 7:**
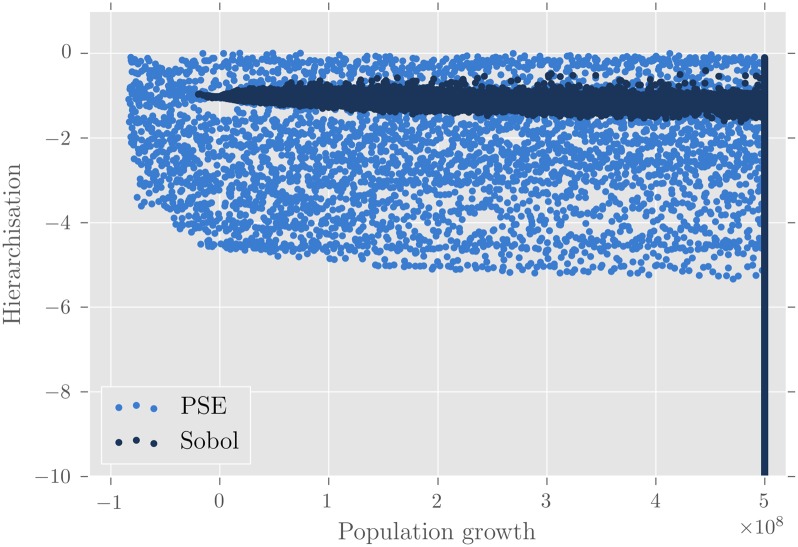
Patterns discovered by PSE and the Sobol sampling for the model MARIUS. Only one replication of PSE is shown. Both methods were run for 3.5 million model evaluations. Each blue dot corresponds to a pattern. PSE found a more diverse range of patterns than the Sobol sampling.

During the first long run experiment, a total of 4555 distinct patterns were discovered, and 95% of them were discovered within the first 48000 hours (cumulative time of the simulations running in parallel). The exploration stopped making new discoveries after about 150000 hours (13.5 million model evaluations). Fig 6 shows the evolution of the number of patterns discovered during the second experiment. The comparison between PSE and the Sobol sampling shows that PSE was much more efficient at discovering diverse patterns for the model MARIUS’s (Figs 6 and 7).

**Fig 8:**
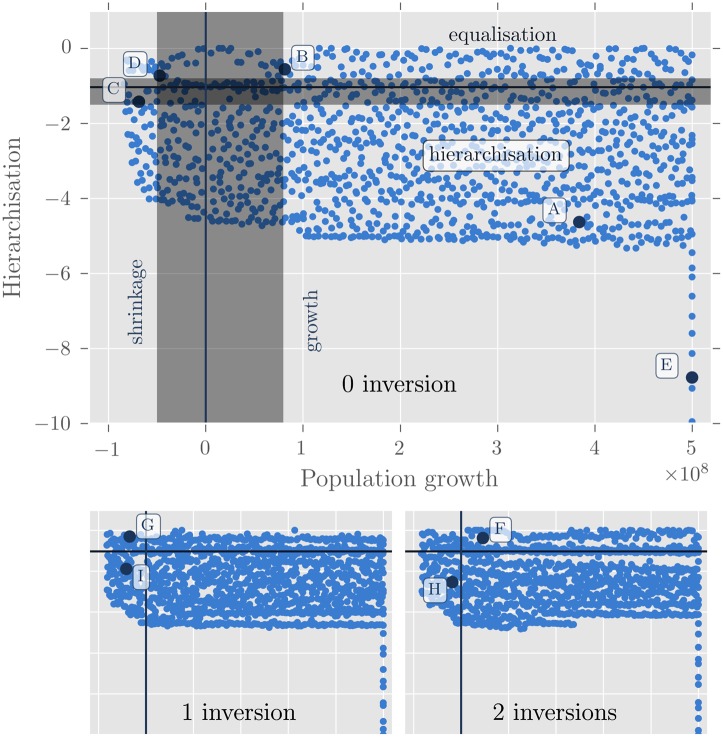
Patterns discovered for the model MARIUS with PSE. The vertical and horizontal lines show the initial state of the system. The grey intervals represent plausible values for each dimension. Letters A-I give the behaviours that are inspected in more details in Table 6, Fig 9 and in the Results section.

The patterns found during the long run exploration are presented in more details in Fig 8 and analysed below. The results are shown for 0 (monotonic population growth), 1 and 2 inversions.

The grey spans correspond to a realistic behaviour of empirical systems of cities with respect to the total growth of urban population (5% per year being the maximum observed in recent China, [48, 49]) and to the hierarchy of their size distribution (the maximum empirical range for the exponent of the rank-size distribution in the recent world being comprised between 0.8 in Egypt and 1.5 for 70 countries in the last 30 years [50]).

The dots on the far right which go below a hierarchisation value of -6 correspond to simulations where population growth is greater than 500 million. As explained above, simulation where population growth is greater than 500 million were assumed to have a growth of 500 million, and are shown at this value on the plot (likewise for the hierarchisation which was bounded at -10). Preliminary experiments not shown here revealed that population growth can get far above 500 million, and hierarchisation can get far below -10, and the number of inversions up to 28 (the maximum that can happen in the 30 steps of the simulation).

**Fig 9:**
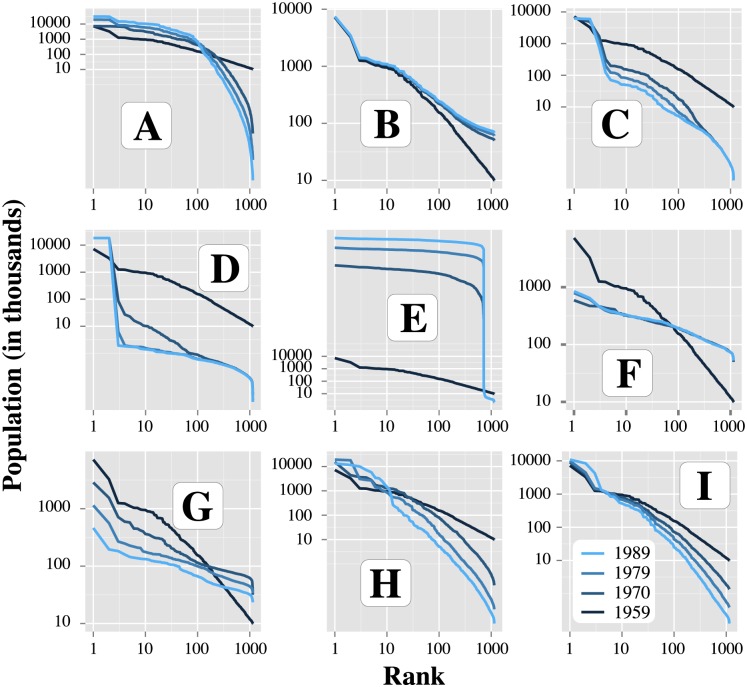
Evolution of the rank-size distributions in the nine inspected simulations of the model MARIUS. The rank refers to the position of the cities sorted by decreasing population size.

Indeed, the 4 kinds of expected behaviours (hierarchisation and growth, hierarchisation and shrinkage, equalisation and growth, equalisation and shrinkage) were found within the empirically plausible bounds. But many patterns were also found to be distributed outside of the grey spans. These patterns are among the potential falsifiers or potential predictions of the model, and are what PSE was designed to discover. They constitute the basis on which the model’s assumptions can be revised. We picked nine and inspect them in more details below. They are labelled from A to I in Fig 8, the evolution of their rank-size distribution is given in Fig 9, and their parameter and pattern values are given in Table 6.

**Table 6:** Parameter (italics) and pattern (bold) values for the 9 inspected patterns.

	A	B	C
*economicMultiplier*	0.002108	0.248015	0.004238
*sizeEffectOnSupply*	1.535866	1.392815	1.911325
*sizeEffectOnDemand*	1.088266	1.180051	1.223311
*wealthToPopulationExponent*	0.615699	0.228982	0.494603
*distanceDecay*	2.516997	0.395674	0.859162
*populationToWealthExponent*	1.770787	1.016049	1.605315
*bonusMultiplier*	0.122952	169.159662	0.080951
*fixedCost*	0	0	0.289442
**Rank-size slope**	-4.62	-0.56	-1.42
**Population difference**	383 × 10^6^	81 × 10^6^	-68 × 10^6^
**Inversion count**	0	0	0
	D	E	F
*economicMultiplier*	0.044799	0.022930	0.199871
*sizeEffectOnSupply*	1.505378	1.659717	1.099736
*sizeEffectOnDemand*	1.173295	1.617497	1.544439
*wealthToPopulationExponent*	0.796531	0.505149	0.759320
*distanceDecay*	0.846435	0.428968	0.798650
*populationToWealthExponent*	1.271591	1.118053	1.348373
*bonusMultiplier*	0	412.164462	0
*fixedCost*	0.021403	0.791581	0
**Rank-size slope**	-0.73	-8.77	-0.38
**Population difference**	-47 × 10^6^	500 × 10^6^	46 × 10^6^
**Inversion count**	0	0	2
	G	H	I
*economicMultiplier*	0.009592	0.247120	0.000592
*sizeEffectOnSupply*	1	1.141378	1.579275
*sizeEffectOnDemand*	1.114942	1.069043	1.141962
*wealthToPopulationExponent*	0.776741	0.884321	0.463994
*distanceDecay*	0.672053	0.725789	0.710989
*populationToWealthExponent*	1.356863	1.023220	1
*bonusMultiplier*	0.082234	0	0.112958
*fixedCost*	0.000747	0	0.000949
**Rank-size slope**	-0.31	-2.54	-1.90
**Population difference**	-33 × 10^6^	-18 × 10^6^	-41 × 10^6^
**Inversion count**	1	2	1

Patterns A and B contrast mainly in the behaviour exhibited by small cities (Fig 9). In A, hierarchisation is performed by their continuous loss of population in favour of the 100 largest cities, while in B, on the contrary, growth of total urban population is due exclusively to the rise of small cities, resulting in an unprecedented equalisation pattern. The distribution of growth with relation to size is controlled by the parameter *wealthToPopulationExponent*, 0.62 in A and 0.23 in B, meaning that the same gain of wealth in two cities attracts more people to the smallest cities in both configurations, but to a larger extent in B, resulting in this equalisation of city sizes. Pattern A represents an unlikely configuration because hierarchisation is usually achieved by a relatively higher growth rate for large cities, and not through a dramatic shrinkage of the small ones as in A. B is less unlikely but it goes against recent observation of a larger attraction of cities with large populations. It could be a prediction of evolution in a context of high costs of congestion and preference for small urban clusters.

In a context of shrinkage, pattern C and D both appear very unlikely, at least, in such a short period of time. First of all, those extreme simulations correspond to a loss of half the system’s population in 30 years. C corresponds to a shrinkage process coupled with hierarchisation, while in D, cities are less differentiated on average as the simulation goes. In both patterns, the majority of cities experience a loss in their population equivalent to one or two orders of magnitude within the first eleven years (1959–1970), and then stagnate around this value. This process is very unrealistic. These patterns must be considered falsifiers of the model. The model assumptions responsible for them need to be re-examined. On the other hand, patterns C and D appear similar in the way the two largest cities (Moscow and Saint Petersburg in the initialisation) end the simulation in a macrocephalic position. This suggestion of resilience for the largest cities of a system is consistent with recent observed trends in shrinking city systems and prospective scenarios of shrinkage management [43].

Finally, in a scenario of continuous growth (with no inversion), Pattern E appears as a most unrealistic extreme case. It results from the very high value of the *bonusMultiplier* parameter (412), that over-values any exchange taking place with another city with respects to the production value. This is an example of the pattern exploration highlighting unreasonable parameter bounds.

Pattern F to I involve one or two inversions of growth. We explored these patterns for prospective purposes, since reversion of urban growth has happened twice in Russia during the last twenty years. By exploring those patterns, we aim at evaluating the interactions between reversion of growth and hierarchisation. Those four patterns show that growth inversions have minor influence on the overall patterns (in F and G: equalisation of city sizes and in H and I: hierarchisation).

F and H have in common two growth inversions and the deactivation of the exchange criteria (*bonusMultiplier* and *fixedCosts* = 0), which means that an exchange within the city has the same value as an interurban exchange. The reduction of the model structure by muting two mechanisms could explain the variability of growth and mimics cities’ localism observed empirically during the 1990s and 2000s in Russia. However, pattern H seems a lot more reasonable than F in terms of the continuity of the (de)hierarchisation trend.

In G, shrinkage is stronger for the largest cities while the smallest ones continue to grow. This configuration is obtained by making consumptive behaviour superlinear (*sizeEffectOnDemand* = 1.11) and productive behaviour linear (*sizeEffectOnSupply* = 1) with city size. The advantage given to urban aggregates of low population results in the loss of attractivity (and population) of cities above 100,000 inhabitants and the growth of cities below that point. This configuration is quite unlikely in the present conditions, where agglomeration economies and integration in the global networks are decisive of city growth, but constitutes an attractor to be considered.

Finally, pattern I seems a possible pattern to be observed in the future. In this configuration, a couple of large cities maintain a growing evolution in an overall shrinking context. The smaller the city, the more it shrinks. This realistic result is obtained with quite unlikely values for parameters (scaling exponent of production *sizeEffectOnSupply* > 1.5 and linear distribution of wealth with regards to population: *populationToWealthExponent* = 1). Patterns G and I could represent (extreme) stylised scenarios to be explored if one was interested in simulating the evolution of the Former Soviet Union in a prospective manner, because they correspond to two regimes of relationship between cities and the economy: one in which large cities have a clear disadvantage in producing and exchanging, and one in which they are the economic winner of the population distribution. Those scenarios could represent the extent to which congestion costs over- or under-compensate for agglomeration economies in a system of cities experiencing shrinkage.

The analysis above should be seen as one step, unexpected patterns inspection, from the wider iterative process mentionned in the introduction. The methodology we propose is to start with exploring the model’s behaviour as widely as possible, and to use the extreme patterns that are found to detect problems in the model’s assumptions and revise them. This is why the parameters bounds in Table 4 are very wide and may seem unrealistic. Rather than trying to guess some reasonable bounds a priori and run the exploration within them, we are trying the opposite approach: guess what bounds are reasonable based on what patterns we observe. The reason for this is that with complex systems, we may have data about the emerging patterns but not so much about the internal mechanisms, and this is the case with city systems (it is easier to know the population size in time through censuses than quantities related to MARIUS’s parameters). The very purpose of complex systems modelling is sometimes to capture internal mechanisms that are not directly observable. In such case, it is easier to say if an observed global pattern is realistic rather than if a parameter value is. This experiment should be seen as just one step of the incremental work aiming at gradually revising the model’s assumptions (both in the model’s internal mechanisms and the parameter bounds) based on the unexpected patterns observed in simulation. Their interpretation above suggests at least that some of the parameters bounds should be revised and the exploration performed again. The continuation of this iterative process will be done in future work focusing on the analysis of the MARIUS model rather than on the PSE algorithm.

## Conclusion

Following Popper’s argument about testing the validity of a scientific theory, we argued in the introduction that looking for unexpected patterns a model can produce, its potential falsifiers, is an important part of the modelling process usually left aside. It has two benefits: to give the modellers the opportunity to revise their model by confronting it to empirical knowledge, and to reveal the model’s predictions.

To discover the unexpected patterns a model can produce, we presented an evolutionary method based on Novelty Search which explores the parameter space of a model looking for diversity of the output patterns, named Pattern Space Exploration or PSE. We tested the method on a model of the well studied phenomenon of collective motion, and then applied it to a model of system of cities, looking at the relationship between city hierarchisation and population growth.

During the tests on the collective behaviour model, the method successfully found the five main expected classes of behaviours: gas, moving liquid, moving solid, stationary liquid and stationary solid. The method’s efficiency to discover a wide range of diverse patterns was compared to the a priori samplings LHS, Sobol, and a regular grid. The results of these comparisons are clearly in favour of the method we propose. It suggests that sampling the parameter space, even with methods that have good space filling properties such as LHS and Sobol’s, is not a good strategy to explore the pattern space generated by a model. Driving the exploration directly in the pattern space, with a method like the one we propose here, is better suited.

The purpose of the experiment with the model MARIUS was to find out what kind of evolution of a system of cities the model could produce, in terms of city hierarchisation and population growth. We found that all combinations of hierarchisation and equalisation on the one hand, and population growth and shrinkage on the other, could be exhibited by the model. We also found that the model predicted values of hierarchisation and equalisation lying far beyond the intervals commonly found in census data. The discovery of unexpected patterns is the core contribution of the method. They can either be counter-examples or predictions of the model. A detailed study of how and why the model produces these patterns is an good opportunity to either revise the model, or make interesting predictions.

While motivated by model falsification to build robust models, this work focuses on the presentation of an algorithm to explore widely the pattern space of a model and on showing experimentally its efficiency in discovering various patterns. Such an exploration is key to discovering the model’s unexpected patterns which serve as a basis for falsification. Future work with the model MARIUS will focus more on using PSE for the iterative process of model falsification and revision for robust model building.

One could think of several ways to achieve the search for diversity in the pattern space with an evolutionary algorithm. We experimented with the one which seemed the simplest: at the selection stage, select the individuals whose behaviours were the least encountered. Other alternatives come to mind. In particular, one could think of selecting the individuals which when used for replication through crossover and mutation produce offsprings with the most diverse behaviours. Such alternatives may constitute directions for future research.

The incentive for the development of PSE is to provide tools for good modelling practice and robust model development. We hope this paper will attract modellers’ attention on an important flaw in today’s common model validation method based on corroboration. Robust model design must also rely on falsification. The PSE method is a proposition to help modellers discover both potential flaws and predictions of their models.

## Supporting Information

S1 Appendix MARIUS ODD description. (PDF)

S1 Fig Patterns discovered for each PSE replication. Rows show the patterns discovered by each replicated run of PSE using the flocking model. See also caption of Fig 4.
(PNG)

S2 Fig Patterns discovered for each LHS replication. Idem, with the LHS a priori sampling.
(PNG)
