# Supplementary material for: Beyond Corroboration: Strengthening Model Validation by Looking for Unexpected Patterns
Source: PLoS One. 2015 Sep 14;10(9):e0138212. doi: 10.1371/journal.pone.0138212 (PMC4569327; doi:10.1371/journal.pone.0138212)
Supplement: S1 Appendix — (PDF) [file pone.0138212.s001.pdf]

ODD description of MARIUS
01/16/15

Disclaimer : *The following ODD presentation implies that MARIUS is seen through the ABM paradigm, where agents are cities, spatial entities instead of individuals. Therefore, social and cognitive aspects of the description of MARIUS will be quite loosely defined, if ever.*

1. Overview

1.1. Purpose
This model targets a specific system in a specific time-span : the Soviet system of cities, from 1959 to 1989. It is grounded on the evolutionary theory of system of cities, and intends to identify the minimal set of interaction mechanisms, able to explain some stylised facts (or patterns) observed in the actual system. Patterns refers to structural characteristics commonly used by geographers to qualify systems of cities : their hierarchization, the spacing of cities among the territory, and cities functional differentiation (see section 1), whereas interactions mechanisms intend to model repeated exchanges between the cities (of goods, services, information and persons).

1.2. Entities , State variables , Scale
The system is made of 1145 cities of more than 10 000 inhabitants in 1959, localised via actual latitudes and longitudes in former Soviet Union (fig.1)

Figure 1 : Empirical distribution of cities in the Soviet Union : 1959-1989

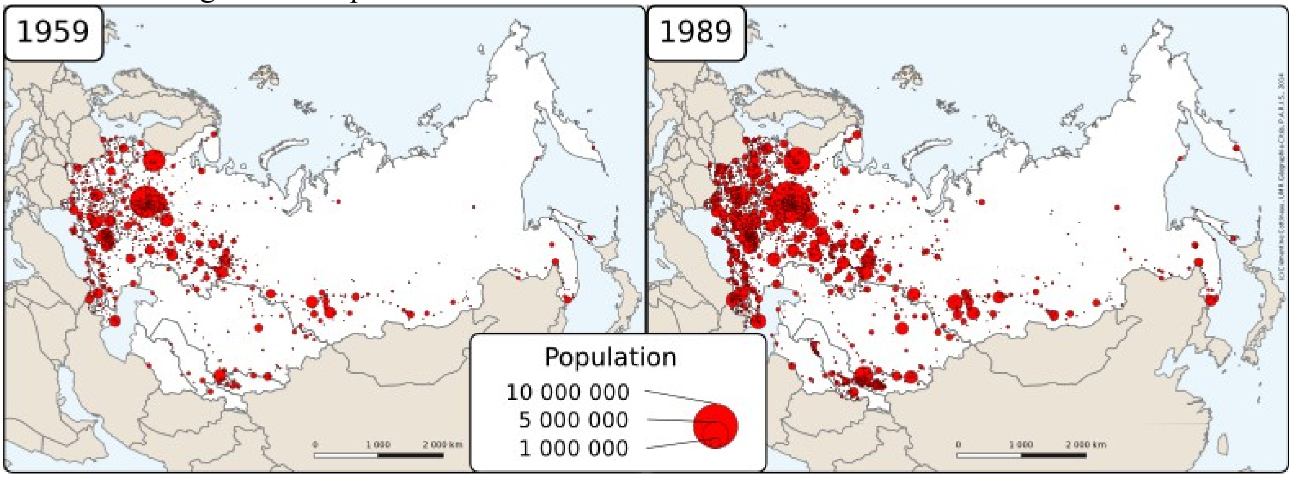

Source : DARIUS Database. Cottineau, 2014. DOI : http://dx.doi.org/10.6084/m9.figshare.1108081

There is only one type of agent : urban agglomerations (we will use the shorter term *city* henceforward), who are characterised by a unique low-level variable to be confronted with empirical data : their *population*. From this single variable are derived other economic variables, involved in the interactions mechanisms :

- wealth
- supply
- demand

In the final stage of development presented in this paper , one more low level variable is computed for each city : TotalOverflowRatio.
Observed State variables includes, but are not limited to, the total urban growth, the rank-size distribution shape of the simulated cities, and the diversity of their trajectories

1.3. Processes overview and scheduling

Time is modelled as discrete steps, each of which representing a time period of one year, during which interactions occur in a synchronous way.

At each step :

1. each city updates its economic variables according to its population (supply, demand).
2. each city interacts with the others according to the intensity of their interaction potential. For two distinct cities, A and B, an interaction consists in confronting A's supply to B's demand, resulting in a transaction of goods sent from A to B.
3. each city updates its wealth according to the results of the transactions in which it was committed.
4. each city updates its population according to its new resulting wealth

2. Design concepts

Emergence : The desired phenomonon to emerge is the diversity of cities trajectories (observed at the whole system scale) and the upkeep of the hierarchisation in the population distribution. Hierarchy itself is pre-existent as it is introduced via the census data of 1959.

Adaptation : Adapatation occurs when the outcomes of transactions betwen cities (gain or loss of wealth) are reported on cities' population (stage 8 of process overview).

Objectives, Learning, Prediction : In the simple model presented in this paper, there is no cognitive dimension in the behaviour of cities.

Sensing : Hypotheses are made that each city is able to know the demand, the supply and the localisation of every other city in the system.

Interactions : The only interactions between cities in this simple model are the confrontation of supplies and demand, resulting in transactions. This confrontation is framed by a gravity model , defining a asymmetric matrix of interaction potential based on supplies and demands of cities, and distances separating them.

Stochasticity, Collectives : There is no stochasticity nor collectives considerations in this generic model.

Observation : Basically, the whole population of the system is observed at each time step. The main focus of observation is set on the population distribution, over time, for the 1145 cities of the system (for a comparison purpose with empirical data, to obtain calibration error) . We also monitor synthetic variable distributions (wealth, supply and demand), especially to highlight ruined cities or over-producing/ over-consuming cities.

Initialization : Population of each city is initialized with the population registered in the census of 1959.

Input Data : In this version, of the model does not use input data to represent time-varying processes.

3. Details

3.1. Sub-Models :

Here are the details of the four points of the process overview.
Equations are numbered like in the paper.

- **each city updates its economic variables according to its population (supply, demand).**

The wealth attribute of each city is computed via th relation of Equation 2, resulting in a superlinear distribution of wealth.

Eq.2 : $wealth_i = population_i^{populationToWealthExponent}$

Supplies and Demands are computed likewise, via a scaling exponent expressing the effect of the size of the city on its productivity and consumption per inhabitant.

Eq.3 :
$$Supply_i = EconomicMultiplier * population_i^{sizeEffectOnSupply}$$
$$with\ EconomicMultiplier > 0$$
$$with\ sizeEffectOnSupply \geq 1.$$

Eq.4 :
$$Demand_i = EconomicMultiplier * population_i^{sizeEffectOnDemand}$$
$$with\ EconomicMultiplier > 0$$
$$with\ sizeEffectOnDemand \geq 1$$

- **each city interacts with the others according to the intensity of their interaction potential. For two distinct cities, A and B, an interaction consists in confronting A's supply to B's demand, resulting in a transaction of goods sent from A to B.**

Interaction potential between two cities i and j is computed following Equation 6

Eq.6 : $$InteractionPotential_{ij} = \frac{Supply_i * Demand_j}{d^{distanceDecay}}$$

Interurban exchange of values

A city i attributes a share of its supply S_{ij} to a city j that is proportional to the share of the interaction potential F_{ij} in the total interaction potential of the city i :

Eq.7 : $$S_{ij} = Supply_i * \frac{F_{ij}}{\sum_k F_{ik}}$$

Symetrically, a city i attributes a share of its demand D_{ij} to a city j that is proportional to the share of the interaction potential F_{ji} in the total interaction potential of the city i :

Eq.8 : $$D_{ij} = Demand_i * \frac{F_{ji}}{\sum_k F_{ki}}$$

The effective transaction from a city i to a city j is determined as :

Eq. 9 : $Transacted_{ij} = min(S_{ij}, D_{ji})$

- **each city updates its wealth according to the results of the transactions in which it was committed.**

Wealth is updated acording Equation 10

Eq.10 :
$$Wealth_{t,i} = Wealth_{t-1,i} + S_i - D_i - unsold_i + unsatisfied_i$$
$$with\ unsold_i = Supply_i - \sum_j Transacted_{ij}$$
$$with\ unsatisfied_i = Demand_i - \sum_j Transacted_{ji}$$

- **each city updates its population according to its new resulting wealth**

The variation of pupulation is computed following Equation 11

Eq.11 : $$\frac{\Delta Population_i}{\Delta t} = \frac{wealth_{i,t}^{wealthToPopulationExponent} - wealth_{i,t-1}^{wealthToPopulationExponent}}{EconomicMultiplier}$$

Then the population is updated

Eq.12 : $$population_{i,t} = population_{i,t-1} + \frac{\Delta Population_i}{\Delta t}$$
