# Supplementary figures and images for: Beyond Corroboration: Strengthening Model Validation by Looking for Unexpected Patterns
Source: PLoS One. 2015 Sep 14;10(9):e0138212. doi: 10.1371/journal.pone.0138212 (PMC4569327; doi:10.1371/journal.pone.0138212)

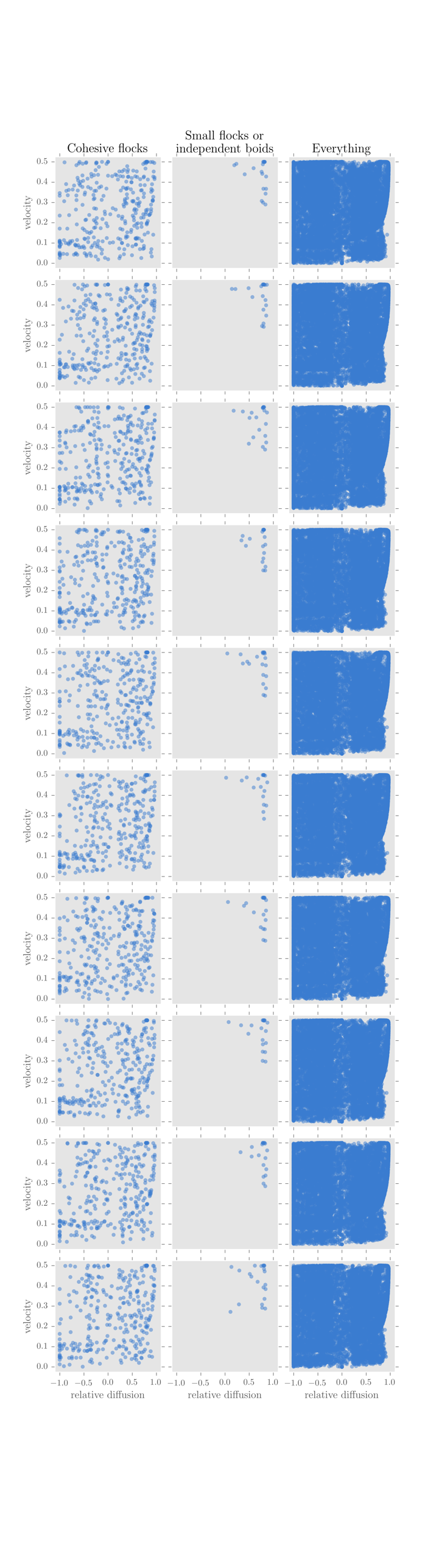

Supplement: S1 Fig — Rows show the patterns discovered by each replicated run of PSE using the flocking model. See also caption of Fig 4. (PNG) [file pone.0138212.s002.png]

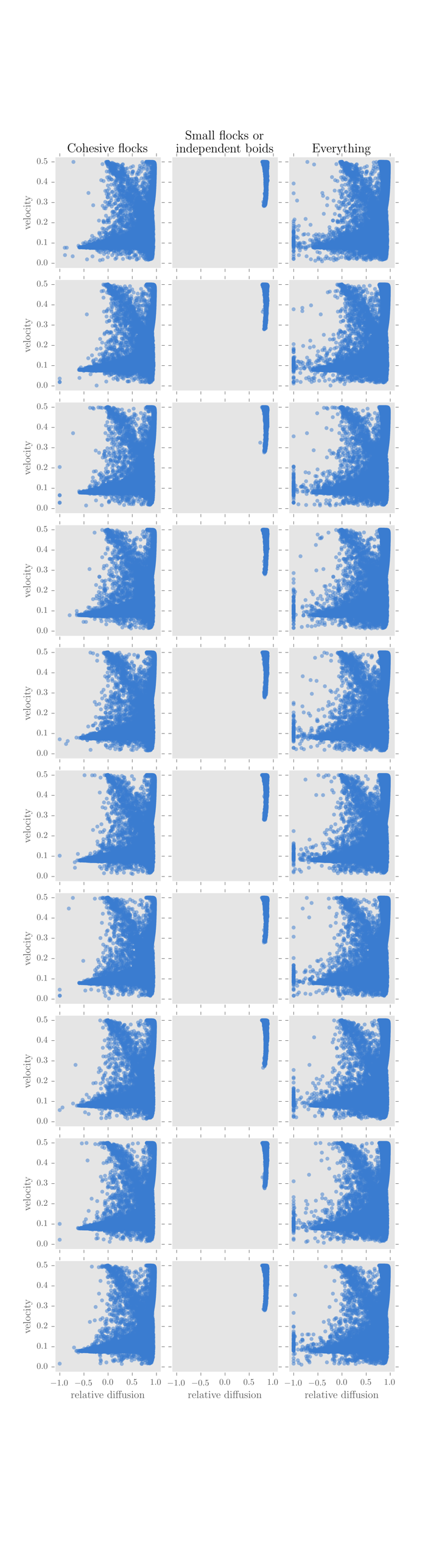

Supplement: S2 Fig — Idem, with the LHS a priori sampling. (PNG) [file pone.0138212.s003.png]
